# Successful Case of Cæsarian Section

**Published:** 1826-07

**Authors:** 


5. *Cæsarian Section.* Dr. Vonderfuhr, of Dahlen was called to a woman, in her first child-bed on the 28th April, 1823, whom he found feeble, emaciated, and rachitic. She was 31 years of age, and had been three days in labour, the waters having been discharged the preceding day. The malformation of the pelvis was evident, the pubes being strongly pressed inwards, and the conjugate diameter not quite two inches. The head of the child was closely impacted—the labour pains severe and at short intervals—child alive. The Cæsarian section was proposed and assented to. The operation was performed, with the assistance of Dr. Kopstadt and Mr. Buckling. The linea alba was the part selected for the incision, which was carried from near the umbilicus to within an inch of the pubes. The uterus being laid bare, was also opened to the extent of five or six inches. The fœtus was extracted with some difficulty. No hæmorrhage ensued. The placenta was extracted next, and the uterus contracted immediately. The patient never uttered a syllable during the whole operation! Sutures and adhesive straps were applied. On the 5th day the operator found the patient remarkably well—free from fever—lochia discharging per vaginam—and milk in the breasts. On the 8th day the wound, excepting a very small portion, was united. She perfectly recovered without a bad symptom. She brought up her infant safely. It is supposed, and not without reason, that much of the success of this terrible operation was owing to the tranquillity of mind and philosophic heroism of the patient. It is, indeed, astonishing with what firmness the softer sex bear the most painful operations. They certainly have more real practical philosophy than boasted man, and deserve a better fate than Providence has allotted them in this world.

